# *KRAS* Mutations in Colorectal Adenocarcinoma: Incidence and Association with Histological Features with Particular Reference to *Gly12Asp* in a Multicenter GIPAD Real-World Study

**DOI:** 10.3390/cancers17172721

**Published:** 2025-08-22

**Authors:** Paola Parente, Valentina Angerilli, Federica Grillo, Maria Raffaella Ambrosio, Federica Petrelli, Jessica Gasparello, Francesca Antoci, Emanuela Pilozzi, Stefania Scarpino, Flavia Adotti, Andrea Ascione, Norman Veccia, Alessandro Caputo, Mariantonia Giobbe, Roberta Gafà, Laura Melocchi, Laura Gandolfi, Paola Parrella, Barbara Pasculli, Francesco Vasuri, Maria Cristina Macciomei, Alessandro Vanoli, Luca Saragoni, Giovanni Lanza, Luca Mastracci, Matteo Fassan

**Affiliations:** 1 Pathology Unit, Fondazione IRCCS Ospedale Casa Sollievo Della Sofferenza, Viale Cappuccini, 71013 San Giovanni Rotondo, Italy; 2 Surgical Pathology Unit, Department of Medicine, University of Padua, Via Gabelli 60, 35121 Padua, Italy; valentina.angerilli@aulss2.veneto.it (V.A.); jessica.gasparello@unipd.it (J.G.); 3 Surgical Pathology Unit, ULSS2 Marca Trevigiana, Piazzale Ospedale 1, 31100 Treviso, Italy; matteo.fassan@unipd.it; 4 Anatomic Pathology Unit, Department of Surgical Sciences and Integrated Diagnostics (DICS), University of Genova, Via Balbi 5, 16026 Genova, Italy; federica.grillo@unige.it (F.G.); luca.mastracci@unige.it (L.M.); 5 Ospedale Policlinico San Martino, IRCCS for Oncology and Neuroscience, Largo Benzi 10, 16132 Genova, Italy; 6 Azienda Ospedaliera Universitaria “SS. Annunziata”, University of Calabria (UNICAL), Via Migliori 1, 87100 Cosenza, Italy; mariaraffaella.ambrosio@unical.it; 7 UOC Anatomia Patologica, Azienda Toscana Nord Ovest, Via Cocchi, 56121 Pisa, Italy; federica.petrelli@uslnordovest.toscana.it; 8 SC Anatomia Patologica, Fondazione IRCCS Policlinico San Matteo, Via Carlo Forlanini 14, 27100 Pavia, Italy; francesca.antoci@unipv.it (F.A.); alessandro.vanoli@unipv.it (A.V.); 9 Department of Clinical and Molecular Medicine, “Sapienza” University of Rome, UOC Anatomia Patologica Morfologia e Molecolare Azienda Ospedaliera-Universitaria Sant’Andrea, Via di Grottarossa 1035, 00189 Roma, Italy; emanuela.pilozzi@uniroma1.it (E.P.); stefania.scarpino@uniroma1.it (S.S.); 10 Department of Experimental Medicine, Sapienza University of Rome, Viale Regina Elena, 324, 00161 Rome, Italy; flavia.adotti@uniroma1.it (F.A.); andrea.ascione@uniroma1.it (A.A.); 11 UOC Anatomia Patologica Azienda Ospedaliera San Camillo Forlanini, Circonvallazione Gianicolense 87, 00152 Roma, Italy; nveccia@scamilloforlanini.rm.it (N.V.); mmacciomei@scamilloforlanini.rm.it (M.C.M.); 12 Department of Medicine, Surgery and Dentistry ‘Scuola Medica Salernitana’, University of Salerno, Via S. Allende 43, 84081 Baronissi, Italy; alcaputo@unisa.it (A.C.); m.giobbe@studenti.unisa.it (M.G.); 13 Department of Pathology, University Hospital of Salerno, Largo Città d’Ippocrate 1, 84131 Salerno, Italy; 14 Anatomic Pathology Unit, Department of Translational Medicine, University of Ferrara, Azienda Ospedaliero-Universitaria S. Anna, Via Aldo Moro, 8, 44124 Ferrara, Italy; roberta.gafa@unife.it (R.G.); lng@unife.it (G.L.); 15 Pathology Unit, Fondazione Poliambulanza Instituto Ospedaliero, Via Leonida Bissolati 57, 25124 Brescia, Italy; laura.melocchi@poliambulanza.it (L.M.); laura.gandolfi@poliambulanza.it (L.G.); 16 Laboratory of Oncology Fondazione IRCCS Ospedale Casa Sollievo Della Sofferenza, Viale Cappuccini, 71013 San Giovanni Rotondo, Italy; pparrella@operapadrepio.it (P.P.); b.pasculli@operapadrepio.it (B.P.); 17 Pathology Unit, Santa Maria delle Croci Hospital, Ravenna, Viale Randi 5, 48121 Ravenna, Italy; francesco.vasuri2@unibo.it (F.V.); luca.saragoni7@unibo.it (L.S.); 18 Department of Medical and Surgical Sciences (DIMEC), University of Bologna, Via Inerio 49, 40126 Bologna, Italy; 19 Anatomic Pathology Unit, Department of Molecular Medicine, University of Pavia, 27100 Pavia, Italy; 20 Veneto Institute of Oncology, IOV-IRCCS, Viale Gattamelata, 35121 Padua, Italy

**Keywords:** biomarkers, colorectal adenocarcinoma, *Gly12Asp*, *KRAS*, mutational analysis, p.G12D

## Abstract

Introduction: Colorectal cancer (CRC) is the third most frequent malignancy and the second cause of cancer-related death worldwide. CRC is characterized by morphologic and biological heterogeneity, and molecular profiling is required to select appropriate treatment in the metastatic setting. Mutations in *KRAS* are detected in approximately 40% of CRCs, with prognostic and predictive value, and with the most frequent being p.G12D. Nonetheless, there are few data on the morphologic features in *KRAS*-mutated CRCs. Materials and Methods: We retrospectively collected clinicopathological features and molecular profiles of CRCs in a multicenter cohort. Results: A total of 2816 patients from 12 centers were included. *KRAS* mutation was found in 47.4% of cases; *Gly12Asp* was detected in 23.9%, with different mutation frequencies between centers. Clinicohistological features associated with *Gly12Asp* mutation included younger patient age (≤70 years of age), higher prevalence in males (58.6%), NOS histotype (87.1%), low pathologic grade (73.9%), high grade budding—Bd3 (43.8%), and tumoral lympho-vascular invasion (68.9%). Conclusions: Recent data have pinpointed the prognostic and predictive value of *Gly12Asp* mutation, and our results contribute to understanding its biology, with particular focus on peculiar clinicopathological features. Moreover, we found significant differences in pathology reports and assays for molecular profiling in different centers, which can affect a standardized therapeutic approach in CRC.

## Simple Summary

Colorectal cancer (CRC) is the third most frequent malignancy and the second cause of cancer-related death worldwide, and it is characterized by a complex molecular landscape involving several genes. Mutations in *KRAS* are the most frequent, present in approximately 40% of CRCs, with a well-recognized predictive value of resistance to anti-EGFR monoclonal antibodies. Moreover, *KRAS* is characterized by a wide range of mutations, among which the most frequent are *Gly12Cys* (p.G12C) and *Gly12Asp* (p.G12D). There are few data concerning the morphologic features associated with these mutations in CRC. Here, we aim to investigate histologic findings in a multicenter cohort of CRC patients, with particular reference to the *Gly12Asp* mutation.

## 1. Introduction

Colorectal cancer (CRC) is the third most frequent malignant neoplasm and the second leading cause of cancer-related death in the United States [1,2]. CRCs are characterized by heterogeneity in both pathogenesis and molecular pathways, leading to different histological features, incidence, sidedness, and outcomes [3]. Many environmental factors are involved in CRC development, such as diet, age, and genetic factors like Lynch syndrome [4].

Histologically, 90–95% of all CRCs are adenocarcinomas, further subclassified according to the 5th Edition of the WHO Classification of Gastro-intestinal Tumors into different subtypes, such as adenocarcinoma not otherwise specified (NOS), micropapillary carcinoma, mucinous adenocarcinoma, serrated adenocarcinoma, medullary adenocarcinoma, and undifferentiated carcinoma [5]. Other rare histotypes have also been described [6].

Mutations in *KRAS* are the most frequent in CRC, affecting approximately 30% to 40% of cases [7,8]. Importantly, *KRAS* mutations in CRC have prognostic and predictive value [9]. In particular, shorter overall survival (OS) has been associated with mutations in codons 12 and 61 [10], whereas mutations producing abnormal smaller K-Ras proteins, leading to fewer binding sites and to rapid and tight bonds to GTP in its active form, cause anti-EGFR drug resistance [11]. In this context, identifying *RAS* mutational status is mandatory for all stage IV CRC patients to select *KRAS* wild-type (wt*KRAS*) patients that can benefit from anti-EGFR treatment, according to ASCO [12] and ESMO [13] guidelines. Moreover, a recent subgroup analysis of the Keynote 177 trial showed a differential effect of anti-PD1 therapy according to *RAS* mutational status, suggesting a lack of benefit among patients with mutated *KRAS* (m*KRAS*) CRC [14].

Recent findings have pinpointed the association of two *KRAS* mutations, *Gly12Cys* (p.G12C) and *Gly12Asp* (p.G12D), with peculiar clinical features and therapeutic options. In particular, *KRAS Gly12Cys* mutation is associated with poor OS, although it has been identified as a druggable target and possible predictor of response to the AMG510 drug [15,16]. The prognostic and predictive value of *KRAS Gly12Asp* mutation, on the other hand, is a matter of debate [17]. The impact of both of these molecular features on the histologic aspects of CRC is an ongoing subject of research.

The current study aims to investigate the incidence of *KRAS* mutations in a multicenter cohort of patients with CRC and their association with clinicopathological features, with specific consideration of the characterization of m*KRAS Gly12Asp* CRCs.

## 2. Materials and Methods

### 2.1. Clinicopathologic Data

Clinical and pathologic data of all CRCs undergoing molecular profiling from January 2020 to December 2024 were retrospectively collected, regardless of clinical and pathological stage, from the pathological archives of the pathology units belonging to the Italian Group of Digestive Disease Pathology (Gruppo Italiano Patologi Apparato Digerente (GIPAD). In particular, data from the pathology units of the Fondazione IRCCS Casa Sollievo della Sofferenza, Treviso Hospital, University of Genova-Ospedale Policlinico San Martino, University of Pavia-Policlinico San Matteo, University of Ferrara, University of Salerno, Azienda Toscana Nord-Ovest, Azienda Ospedaliera Universitaria Sant’Andrea di Roma, Azienda Ospedaliera Universitaria Umberto I di Roma, Azienda Ospedaliera San Camillo Forlanini di Roma, Fondazione Poliambulanza di Brescia, and University of Bologna-Ospedale Santa Maria delle Croci di Ravenna were collected. All information regarding human tissue was managed using anonymous numerical codes, and all data were handled in compliance with the Declaration of Helsinki.

### 2.2. Pathologic Features

For each case, data on patient’s gender, age at diagnosis and surgery, tumor site, histologic type, grading (for NOS and mucinous histotypes) according to the 5th edition of WHO Classification of Gastro-intestinal Tumors [5], tumor budding (based on the three-tier system according to ITBCC 2016 [18]), tumor vascular invasion, tumor perineural invasion, and pTNM staging according to AJCC [19] were collected.

### 2.3. MMR/MSI Status

Nuclear immunostaining expression for each of the four proteins (MLH1, PMS2, MSH2, and MSH6) of the Mismatch Repair Complex (MMR) was evaluated locally, and details were identified from the original pathology reports. In particular, a case was classified as proficient MMR (MMRp) CRC when retained nuclear immunostaining for all 4 proteins was observed in ≥10% of tumor cells; a case was classified as deficient MMR (MMRd) CRC when loss of expression of one or more proteins was seen in neoplastic cells. Cases showing one or more proteins with retained immunostaining in <10% of tumor cells and/or with neoplastic cells with weaker immunostaining than the internal control were classified as indeterminate MMR (MMRi) CRCs [20].

When available, microsatellite status was also collected and classified as MSI (i.e., microsatellite instability) or MSS (microsatellite stability).

### 2.4. KRAS Mutational Analysis

Data on *KRAS* mutational status, including the type of mutation identified, were collected retrospectively from patient records at the participating centers. Molecular testing was performed locally at each center using validated next-generation sequencing (NGS) panels or polymerase chain reaction (PCR)-based assays, according to institutional protocols. Details of the panels and platforms used are listed below:

- Center 1: Myriapod NGS Cancer Panel (Diatech Pharmacogenetics, Jesi, Italy), sequencing on Illumina platform.
- Center 2: EasyPGX ready *KRAS* (Diatech Pharmacogenetics).
- Center 3: Oncomine DX Target Test (Thermo Fisher Scientific, Waltham, MA, USA), sequencing on Ion Torrent platform.
- Center 4: Oncomine Focus Assay (Thermo Fisher Scientific), sequencing on Ion Torrent platform; Myriapod NGS Cancer Panel (Diatech Pharmacogenetics).
- Center 5: Myriapod Colon Panel (Diatech Pharmacogenetics), sequencing on Ion Torrent platform; Myriapod Colon Status Kit (Diatech Pharmacogenetics) on the MassARRAY system (Sequenom, San Diego, CA, USA).
- Center 6, Center 7: Myriapod NGS Cancer Panel (Diatech Pharmacogenetics), sequencing on Illumina; EasyPGX ready *KRAS* (Diatech Pharmacogenetics).
- Center 8: Oncomine Focus Assay (Thermo Fisher Scientific), sequencing on Ion Torrent platform; EasyPGX ready *KRAS* (Diatech Pharmacogenetics).
- Center 9: RAS Mutation Screening Panel (EntroGen, Woodland Hills, CA, USA); EasyPGX ready *KRAS* (Diatech Pharmacogenetics).
- Centers 10 and 11: Myriapod NGS Cancer Panel DNA (Diatech Pharmacogenetics), sequencing on Illumina platform (San Diego, CA, USA); Idylla *KRAS* Mutation Assay (Biocartis, Mechelen, Belgium).
- Center 12: Idylla *KRAS* Mutation Test (Biocartis).

Although all platforms used targeted clinically relevant regions of the *KRAS* gene, including codons 12, 13, and 61, differences in analytical sensitivity and variant coverage among methods may influence mutation detection. NGS platforms (e.g., Myriapod and Oncomine) generally offer high sensitivity and broader variant coverage, including rare and uncommon substitutions such as p.Gly12Asp (G12D). By contrast, some PCR-based assays, such as EasyPGX or Idylla, are limited to predefined hotspot mutations.

The Idylla *KRAS* Mutation Test and Myriapod Colon Status Kit specifically detect and report p.Gly12Asp (G12D) as a discrete result, among other individual *KRAS* mutations. This enables reliable differentiation of G12D from other codon 12 variants. By contrast, the EasyPGX ready KRAS assay does not differentiate G12D from other codon 12 substitutions, such as G12A or G12V, and reports grouped results (G12X).

Mutation calls were interpreted by qualified molecular biologists/pathologists following each center’s standard operating procedures.

### 2.5. Statistical Analysis

Categorical variables were described using frequency. Associations between categorical data were investigated using the Chi-square test and Fisher’s exact test. All tests of significance were two-tailed, and *p*-values of <0.05 were considered statistically significant. Statistical analyses were carried out using SPSS version 26 (IBM, Armonk, NY, USA).

## 3. Results

### 3.1. Patient Cohort

A total of 2816 patients were included in this study from the 12 participating centers, each comprising surgical, pathology, and medical oncology units. In the majority of cases *KRAS* mutational status was assessed using an NGS-based test (61.2%), followed by a PCR-based test (37.6%), and finally by MALDI-TOF (1.2%).

Overall, 1187 (42.2%) patients were females and 1627 (57.8%) were males. Analysis was performed on endoscopy biopsy samples in 692 (24.6%) patients and on surgical specimens in 2124 (75.4%) patients. The majority of cases were localized in the recto-sigmoidal (44.7%) segment, followed by proximal (37.4%) and distal (17.9%) sites, respectively. The most frequent histotype was NOS (87.2%), followed by mucinous (9.3%) and signet ring (1.1%). Medullary, micropapillary, adenosquamous, undifferentiated, and serrated histotypes were also reported, with <1% frequency. Most cases were low grade (64.2%) compared to high grade (35.8%). Bd1 category for tumor budding was the most frequent (38.0%), followed by Bd3 (33.4%) and Bd2 (28.6%). Tumor lymphovascular invasion was described in 1427 (69.0%) cases and tumor perineural invasion in 947 (47.0%) cases. Stages pT3 (57.4%) for tumor extent and pN1 (39.1%) for lymph node involvement were the most frequent. pMMR/MSS profile was documented in the majority of patients (82.7%).

Concerning clinical information, patient’s age was not available in 1 patient, sex in 2 cases, and site in 116 cases. Regarding pathologic features, histotype was not available in 154 cases, grading in 419 cases, tumor budding in 1426 cases, and tumor vascular invasion in 747 cases, mostly due to analysis being performed on endoscopic biopsies. pT and pN stages were not available in 1249 and 1262 cases (of which 692 were biopsies), respectively, whereas MMR/MSI status was unavailable in 298 cases (of which 115 were biopsies).

A detailed description of the clinicopathological features of the study cohort is reported in Supplementary Table S1.

### 3.2. Clinicopathological Features of KRAS-Mutated CRC

Among the 2816 patients analyzed, 1334 (47.4%) harbored a *KRAS* mutation, whereas 1482 (52.6%) were *KRAS* wild-type. The distribution of *KRAS* mutations varied significantly across the 12 participating centers, with mutation frequencies ranging from 36.1% in Center 1 to 62.0% in Center 11 (*p* < 0.001), highlighting potential regional or methodological differences.

Patients with *mKRAS* CRCs were significantly younger than wtKRAS CRC patients, with 50.1% aged > 70 years versus 54.2% in the wtKRAS group (*p* = 0.031). There was no significant difference in sex distribution between groups.

In biopsy specimens, KRAS-mutated cases were more frequent than KRAS wild-type cases (27.2% vs. 22.2%, *p* = 0.002); moreover, KRAS-mutated cases were more often located in the proximal colon (41.0% vs. 34.3%, *p* = 0.002), whereas wtKRAS CRCs were more frequently located in the rectosigmoid region.

Histologically, the mucinous histotype was more frequent in the *mKRAS* CRC group (10.0% vs. 8.6%), while medullary and undifferentiated histotypes were less frequent (0.2% vs 1.2% and 0% vs 0.6%, respectively), compared to the *wtKRAS* CRC group (*p* = 0.02). No statistically significant differences were documented in other histotypes. Tumor grading differed significantly between *wtKRAS* and *mKRAS* groups, with high-grade morphology more frequent in *wtKRAS* CRCs (40.6% vs. 31.1%, *p* < 0.001).

Tumor budding grade also showed a significant association with *mKRAS* CRCs: Bd3 was more frequent in the *mKRAS* group (39.1% vs. 28.9%), while Bd1 was more common in the *wtKRAS* group (*p* < 0.001). No significant differences were found regarding tumor lymphovascular invasion or tumor perineural invasion.

Pathologic T-stage distribution did not differ significantly between groups. However, *wtKRAS* CRCs were more frequently pN2 (35.0% vs. 29.2%), whereas *mKRAS* CRCs were more often pN1 (*p* = 0.013).

Importantly, MMRd profile or MSI status was significantly less common in *mKRAS* CRCs (7.7%) compared to *wtKRAS* (26.2%) (*p* < 0.001), consistent with previously reported partial mutual exclusivity patterns between *KRAS* mutations and MSI.

All data are reported in Table 1.

### 3.3. KRAS Mutational Landscape

**Figure 1:**
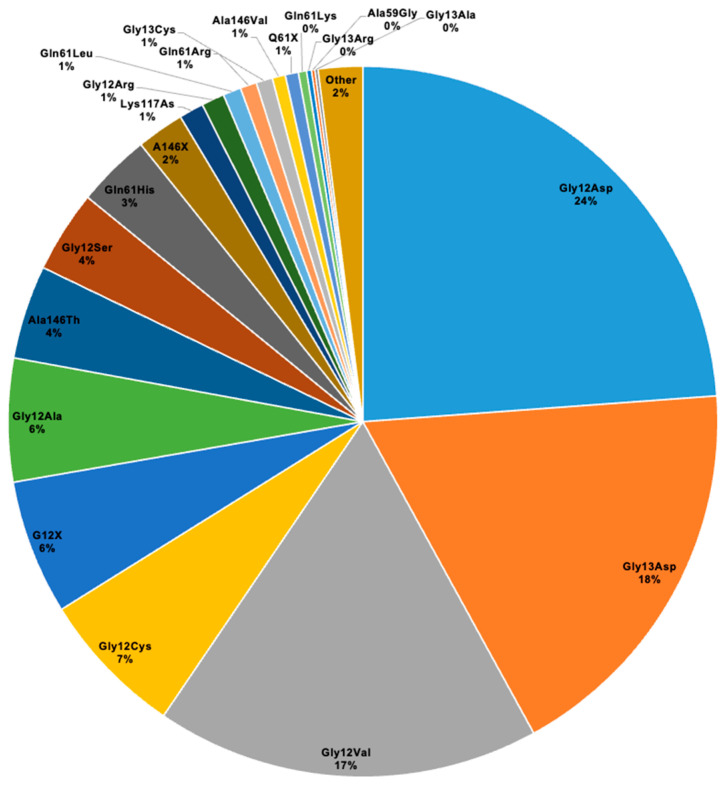
Frequency of *KRAS* mutations.

The four most frequent mutations in *KRAS* were *Gly12Asp* (23.9%), *Gly13Asp* (18.2%), *Gly12Val* (17.5%), and *Gly12Cys* (6.6%) (Table 2A). The frequency of the other *KRAS* mutations is summarized in Figure 1. In eight cases, a double concomitant *KRAS* mutation was documented (*Gly12Asp*+*Gly12Ser*, *Gly12Asp*+*Leu19Phe*, *Gly12Cys*+*Gly12Asp*, *Gly13Asp*+*Ala146Thr*, *Gly13Asp*+*Ala59Gly*, *Ala146Ser*+*Lys147Asn*, *Leu19Phe*+*Leu20Ser*, and *Gly12Ser*+*Gly13Asp*). Aminoacidic substitution was not specified in 121 cases, categorized as G12X (n = 82), A146X (n = 29), Q61X (n = 8), A59X (n = 1), and K117X (n = 1) classes, respectively, due to the inability of some PCR assays to detect the specific nucleotide change.

The distribution of mutations showed some variability across the 12 participating centers, with Center 1 reporting the highest proportions (e.g., *Gly12Asp* 18.5%, *Gly13Asp* 18.5%) (Table 2A). No statistically significant differences in mutation prevalence were observed by patient age (>70 years vs. ≤70 years) or sex (Supplementary Table S2). No statistically significant differences according to specimen type (biopsy vs. surgical) were observed between mutation groups (Supplementary Table S2). Notably, mutation site distribution differed significantly, with *Gly12Asp* and *Gly13Asp* mutations more frequently found in distal/rectosigmoidal tumors (*p* = 0.005 and *p* = 0.03, respectively) (Table 2C). Histological subtype and tumor grading showed modest differences, with *Gly13Asp* mutations more often associated with higher grading (*p* = 0.035) (Table 2D and 2E). Other pathological features, such as tumor budding, tumor lymphovascular and tumor perineural invasion, and pathological T and N stages, did not differ significantly between mutation groups. Detailed correlations between the four most frequent mutations and all clinicopathological features are described in Supplementary Table S2.

### 3.4. Clinicopathologic Features of Gly12Asp-Mutated CRC

Overall, the *Gly12Asp* mutation was more frequent in patients < 70 years of age (52.7%) and in male patients (58.6%). A higher percentage was documented in surgical samples (72.1%) and in low-grade CRC (73.9%). *KRAS* Gly12Asp mCRCs were more frequently located in the rectosigmoid colon (46.8%), in comparison with *KRAS* non-Gly12Asp mCRCs (*p* = 0.005). Tumor budding Bd3 (43.8%) was observed more frequently, followed by Bd2 (30.2%) and Bd1 (26%). Tumor lymphovascular invasion was identified in 68.9% of cases, while tumor perineural invasion was less frequent (45.7%). *Gly12Asp* mutation was found equally in pT3 (47.2%) and pT4 (46%) stage CRCs; *KRAS* Gly12Asp mutation was more frequent in cases with nodal metastases (pN1 39.4% and pN2 34.3%) compared to node-negative cases (pN0 26.3%). Most *KRAS* Gly12Asp mCRCs were MMRp/MSS (93.6%).

Mucinous and serrated histotypes were more frequent in CRCs with *Gly12Asp* mutation compared to the *non-Gly12Asp mKRAS* group (11.2% vs 9.1% and 0.7% vs 0.1%, respectively), whereas no cases of medullary, adenosquamous, and undifferentiated histotype harbored this mutation (Table 2D).

Compared to *Gly12Cys* mutation, the only differences were a higher prevalence of patient age > 70 (59.6%), the presence of tumoral perineural invasion (52.5%), and pN2 stage (48.9%).

## 4. Discussion

CRC is characterized by extensive heterogeneity in terms of etiology, morphology, and molecular landscape. In the metastatic setting, assessment of predictive and prognostic biomarkers is the first step for appropriate treatment choice.

Mutations in *KRAS* are the most frequent, harboring both prognostic and predictive importance. The majority of *KRAS* mutations occur in exon 2, specifically at codons 12 (approximately 51%) and 13 (16%), followed by less frequent mutations in exons 3 (3%) and 4 (6%) [21]. Most of these alterations are single-nucleotide variants leading to amino acid substitutions in codons 12, 13, and 61, which result in increased GTP binding affinity and constitutive activation of RAS signaling [22].

In this setting, recent data have pinpointed two specific *KRAS* mutations, *Gly12Cys* and *Gly12Asp*, as the most interesting for therapeutic purposes. The *Gly12Cys* mutation, with a prevalence ranging from 6% to 17%, is associated with significantly shorter OS compared to other *KRAS* variants (median OS 28.9 vs. 36.7 months) and may predict responsiveness to the targeted inhibitor AMG510 [11,15,16].

*Gly12Asp* mutation has been described as the most frequent *KRAS* mutation in CRC, ranging from 19.2% to 37.5%, with a still debated prognostic and predictive value [9,23]. To the best of our knowledge, no peculiar clinicopathological features associated with *Gly12Asp* mutations have been described in CRC until now.

In this real-world, multicenter study, we aimed to investigate the frequency of *KRAS* mutations in CRC with a particular focus on the clinicopathological characteristics of *Gly12Asp*-mutated CRC.

First of all, we confirmed an overall *KRAS* mutation frequency of approximately 47%, in line with data reported in the Literature [7], and association with the male sex, recto-sigmoidal localization, low-grade, higher pT stage, and MMRp/MSS profile [24]. Discrepancies exist regarding anatomical site on the basis of different studies reporting right or left side predominance [25].

We described a higher prevalence of *KRAS* mutation for the NOS histotype, in contrast to the mucinous histotype, as reported in the literature [6,26]. A higher prevalence of mucinous histotype was documented in surgical specimens compared to biopsies (11.0% vs. 3.2%; *p* < 0.00001), whereas NOS histotype was more frequent in biopsies (95.2% vs. 84.9%; *p* = 0.084762). Of note, almost a quarter of the cases (24.6%) in our cohort were biopsy samples, with intrinsic limitations in histotype evaluation. According to the WHO classification, mucinous histotype is defined when extracellular pools of neoplastic mucin are present in >50% of the whole neoplasm, and this is difficult to evaluate in biopsy specimens.

We confirmed *Gly12Asp* as the most frequent *KRAS* mutation, present in 23.9% of our CRC series, and with reported frequency in recent multicenter studies of 19.2% [9], 37.5% [23], and 33.1% [24], respectively.

Most importantly, for the first time, to the best of our knowledge, we describe the clinicohistological features of CRCs associated with *Gly12Asp* mutation. We documented younger patient age (<70 y.o.; 52.7%) and higher prevalence of male patients (58.6%), NOS histotype (87.1%), low-grade (73.9%), Bd3 (43.8%), tumor lymphovascular invasion (68.9%), and absence of tumor perineural invasion (54.3%). *Gly12Asp* mutation, moreover, had almost the same frequency in pT3 (47.2%) and pT4 (46.0%) stages, respectively, and was most frequent in pN1-2 cases (39.4% and 34.3%, respectively) compared to pN0 (26.3%) cases (Supplementary Table S2). These data require confirmation in selected multicenter series.

In this context, the association with Bd3, tumor lymphovascular invasion, and high pT stage seems to favor a worse prognostic value of *Gly12Asp* mutation, although the lack of clinical data limits our study and interpretation of the results.

From a histologic point of view, the morphologic features of *Gly12Asp* mutation have been described only in lung neoplasms, where an association with invasive mucinous adenocarcinoma histotype has been observed [27].

Finally, and more interestingly, a statistically different frequency of *Gly12Asp* mutation was reported in different centers. This could be explained by the use of different molecular assays and panels. In particular, four centers performed molecular assays with Real Time-PCR EasyPGX as the only test (one center) or in combination with NGS (three centers). Although the RT-PCR assay makes result interpretation easier and is cheaper, it is less comprehensive than NGS and does not allow for discrimination between different variants, thus failing to identify *Gly12Asp* and *Gly12Cys* mutations in *KRAS.* NGS methods, on the other hand, can detect any variant, from single-nucleotide changes to insertions or deletions, in a single multiplex PCR reaction, allowing the identification of uncommon *KRAS* mutations [8]. NGS, however, is more expensive than RT-PCR and requires a higher daily load of tests to reduce turnaround time. For this reason, some laboratories adopt RT-PCR assay instead of NGS [28]. Although economic constraints may influence the choice of different methodologies in laboratories, it would be useful to adopt, when possible, advanced technologies such as NGS in order to be able to offer our patients the best treatment [29].

Of note, some results beyond our proposed aims highlighted important issues to be addressed in clinical practice. In our series, a higher frequency of NOS histotype (87.2% vs 50%) and a lower incidence of micropapillary histotype (0.8% vs 5–20%) were seen compared to the literature [5,6]. Some important pathologic factors were not specified in the pathology reports, including histotype (missing in 154 cases—8.5%), grade (missing in 419 (14.8%) cases), budding (missing in 724 of surgical cases (34%), and MMR/MSI status (missing in 298 (10.5%) cases), even though complete histologic characterization of a neoplasm, according to the WHO, is a fundamental prognostic factor. In CRC, certain histotypes (i.e., micropapillary), high grade, and Bd3 identify poor prognosis, while the MMR/MSI profile has prognostic, predictive, and diagnostic (i.e., Lynch syndrome screening) importance. The lack of these features in a pathology report can impact the therapeutic approach [30]. Incomplete histological characterization, in our series, could be linked to cases undergoing molecular profiling from outside hospitals in molecular hubs, thereby often lacking complete histological reports.

Finally, the anatomical site of CRC was not available in 116 cases (4%), highlighting the need for closer collaboration between clinicians and pathologists to ensure comprehensive and accurate clinical documentation.

Our study has several limitations. The first is the retrospective nature and lack of uniformity in histological reports and molecular assays, limiting a detailed morphological analysis. Second, the lack of clinical data significantly affects the chance to link histological features and disease prognosis, with particular reference to OS. Third, due to the restriction of molecular profiling in the metastatic/recurrent setting, there was no uniform selection of patients, limiting the impact of data on tumor stage and histopathological variables. Last, multivariate analysis was not performed, as the study was primarily descriptive and not designed to assess independent associations.

## 5. Conclusions

In conclusion, our study describes the incidence of *KRAS* mutations in a multicenter, real-world CRC cohort with similar clinicopathological features as reported in the literature and describes, for the first time, the clinicopathological characteristics of CRCs harboring *Gly12Asp* mutations, providing novel insights into their potential biological behavior. Moreover, our results identify significant differences between laboratories in pathology report quality, frequency of CRC histotypes, and molecular assays that can potentially affect a patient’s selection for targeted therapies in CRC.

## Figures and Tables

**Table 1:** Clinicopathological and molecular features of 2816 primary colorectal adenocarcinomas according to *KRAS* status.

		*KRAS mut* (*n* = 1334; 47.4%)		*KRAS wt* (*n* = 1482; 52.6%)		P (Chi-Square)
Center	Center 1	201	15.1%	356	24.0%	<0.001
	Center 2	175	13.1%	187	12.6%	
	Center 3	145	10.9%	163	11.0%	
	Center 4	121	9.1%	170	11.5%	
	Center 5	122	9.1%	108	7.3%	
	Center 6	105	7.9%	93	6.3%	
	Center 7	106	7.9%	84	5.7%	
	Center 8	98	7.3%	90	6.1%	
	Center 9	85	6.4%	100	6.7%	
	Center 10	67	5.0%	56	3.8%	
	Center 11	67	5.0%	41	2.8%	
	Center 12	42	3.1%	34	2.3%	
Assay	NGS-based assay	783	58.7%	939	63.3%	0.011
	PCR-based assay/MALDI-TOF	551	41.3%	543	36.7%	
Age	>70 years	669	50.1%	803	54.2%	0.031
	≤70 years	665	49.9%	678	45.8%	
Sex	M	781	58.5%	846	57.2%	0.458
	F	553	41.5%	634	42.8%	
Specimen	Biopsy	363	27.2%	329	22.2%	0.002
	Surgical	971	72.8%	1153	77.8%	
Site	Proximal colon	522	41.0%	489	34.3%	0.002
	Distal colon	215	16.8%	268	18.8%	
	Rectosigmoid	539	42.2%	669	46.9%	
Histotype	NOS	1096	87.7%	1226	86.8%	0.02
	Mucinous	125	10.0%	122	8.6%	
	Signet Ring cell	9	0.7%	19	1.3%	
	Medullary	3	0.2%	17	1.2%	
	Micropapillary	6	0.5%	14	1.0%	
	Adenosquamous	8	0.6%	4	0.3%	
	Undifferentiated	0	0.0%	8	0.6%	
	Serrated	3	0.2%	2	0.1%	
Grading	High	352	31.1%	514	40.6%	<0.001
	Low	781	68.9%	752	59.4%	
Budding	Bd1	203	33.3%	325	41.6%	<0.001
	Bd2	168	27.6%	230	29.4%	
	Bd3	238	39.1%	226	28.9%	
Lymphovascular invasion	No	293	31.3%	349	30.8%	0.830
	Yes	644	68.7%	783	69.2%	
Perineural invasion	No	502	55.1%	564	51.2%	0.079
	Yes	409	44.9%	538	48.8%	
pT	pT1	6	0.8%	10	1.2%	0.388
	pT2	38	5.0%	54	6.7%	
	pT3	361	47.4%	382	47.3%	
	pT4	356	46.8%	362	44.8%	
pN	pN0	213	28.2%	233	29.1%	0.013
	pN1	321	42.6%	288	35.9%	
	pN2	220	29.2%	281	35.0%	
MMR/MS status	MMRd/MSI	93	7.7%	343	26.2%	<0.001
	MMRp/MSS	1116	92.3%	966	73.8%	

Abbreviations: MMR: mismatch repair; MMRd: mismatch repair deficiency; MMRp: mismatch repair proficiency; MS: microsatellite assay; MSI: microsatellite instability; MSS: microsatellite stability.

**Table 2:** Frequency of the four most frequent *KRAS* mutations according to center (A), Assay (B), anatomical site (C), histotype (D), and grading (E). * n = 82 G12X KRAS-mutated CRCs were excluded.

		Gly12Asp				Gly13Asp				Gly12Val				Gly12Cys			
		Gly12Asp N = 319 (25.5%)		*KRAS* non-Gly12Asp mut * N = 934 (74.5%)		Gly13Asp N = 243 (18.2%)		*KRAS* non-Gly13Asp mut N = 1091 (81.8%)		Gly12Val N = 234 (17.5%)		*KRAS* non- Gly12Val mut * N = 1019 (82.5%)		Gly12Cys N = 89 (6.7%)		*KRAS* non-Gly12Cys mut N = 1245 (93.3%)	
A Center	Center 1	59	18.5%	142	15.2%	45	18.5%	156	14.3%	30	12.8%	171	16.8%	14	15.7%	187	15.0%
	Center 2	29	9.1%	116	12.4%	33	13.6%	112	10.3%	23	9.8%	122	12.0%	9	10.1%	136	10.9%
	Center 3	50	15.7%	123	13.2%	27	11.1%	148	13.6%	42	17.9%	131	12.9%	16	18.0%	159	12.8%
	Center 4	35	11.0%	86	9.2%	20	8.2%	101	9.3%	28	12.0%	93	9.1%	9	10.1%	112	9.0%
	Center 5	30	9.4%	92	9.9%	16	6.6%	106	9.7%	25	10.7%	97	9.5%	6	6.7%	116	9.3%
	Center 6	27	8.5%	78	8.4%	15	6.2%	90	8.2%	19	8.1%	86	8.4%	6	6.7%	99	8.0%
	Center 7	11	3.4%	51	5.5%	20	8.2%	86	7.9%	4	1.7%	58	5.7%	5	5.6%	101	8.1%
	Center 8	9	2.8%	54	5.8%	18	7.4%	80	7.3%	5	2.1%	58	5.7%	5	5.6%	93	7.5%
	Center 9	25	7.8%	60	6.4%	22	9.1%	63	5.8%	13	5.6%	72	7.1%	3	3.4%	82	6.6%
	Center 10	12	3.8%	55	5.9%	10	4.1%	57	5.2%	17	7.3%	50	4.9%	10	11.2%	57	4.6%
	Center 11	21	6.6%	46	4.9%	9	3.7%	58	5.3%	16	6.8%	51	5.0%	3	3.4%	64	5.1%
	Center 12	11	3.4%	31	3.3%	8	3.3%	34	3.1%	12	5.1%	30	2.9%	3	3.4%	39	3.1%
	*p-*value	0.148				0.253				0.008				0.299			
B Assay	NGS-based assay	197	61.8%	584	62.5%	145	59.7%	638	58.5%	144	61.5%	636	62.4%	55	61.8%	728	58.5%
	PCR-based assay/MALDI-TOF	122	38.2%	350	37.5%	98	40.3%	453	41.5%	90	38.5%	383	37.6%	34	38.2%	517	41.5%
	*p-*value	0.806				0.732				0.803				0.538			
C Site	Distal Colon	129	41.9%	393	40.4%	112	48.5%	410	39.0%	74	32.7%	448	42.5%	33	37.5%	489	41.0%
	Proximal Colon	35	11.4%	187	19.2%	34	14.7%	188	17.9%	47	20.8%	175	16.6%	18	20.5%	204	17.1%
	Rectosigmoid Colon	144	46.8%	393	40.4%	85	36.8%	452	43.0%	105	46.5%	432	40.9%	37	42.0%	500	41.9%
	*p-*value	0.005				0.03				0.023				0.678			
D Histotype	NOS	264	87.1%	773	88.3%	201	87.0%	895	87.8%	196	90.3%	841	87.5%	72	87.8%	1024	87.7%
	Mucinous	34	11.2%	80	9.1%	22	9.5%	103	10.1%	19	8.8%	95	9.9%	7	8.5%	118	10.1%
	Signet Ring cell	2	0.7%	6	0.7%	0	0.0%	9	0.9%	1	0.5%	7	0.7%	3	3.7%	6	0.5%
	Medullary	0	0.0%	2	0.2%	0	0.0%	3	0.3%	0	0.0%	2	0.2%	0	0.0%	3	0.3%
	Micropapillary	1	0.3%	5	0.6%	2	0.9%	4	0.4%	0	0.0%	6	0.6%	0	0.0%	6	0.5%
	Adenosquamous	0	0.0%	8	0.9%	5	2.2%	3	0.3%	1	0.5%	7	0.7%	0	0.0%	8	0.7%
	Undifferentiated	0	0.0%	0	0.0%	0	0.0%	0	0.0%	0	0.0%	0	0.0%	0	0.0%	0	0.0%
	Serrated	2	0.7%	1	0.1%	1	0.4%	2	0.2%	0	0.0%	3	0.3%	0	0.0%	3	0.3%
	*p-*value	0.294				0.037				0.965				0.205			
E Grading	High	70	26.1%	235	29.8%	77	37.2%	275	29.7%	46	24.2%	259	29.9%	18	25.4%	334	31.5%
	Low	198	73.9%	553	70.2%	130	62.8%	651	70.3%	144	75.8%	607	70.1%	53	74.6%	728	68.5%
	*p-*value	0.248				0.035				0.117				0.282			

## Data Availability

All data are reported in the text and in the tables.

## Supplementary Materials

The following supporting information can be downloaded at: https://www.mdpi.com/article/10.3390/cancers17172721/s1, Table S1: Clinicopathological and molecular features of 2816 primary colorectal adenocarcinomas from 12 large Italian surgical pathology units; Table S2: *KRAS* mutation frequency according to age (A), sex (B), specimen (C), budding (D), perivascular (E) and perineural (F) invasion, pT (G) and pN (H) stages, and MMR/MS status (L). Table S3: Missing data for variable and relative % of biopsy samples.

## Abbreviations

The following abbreviations are used in this manuscript:

CRC	Colorectal cancer
iMMR	Indeterminate MMR
*mKRAS*	Mutated *KRAS*
MMR	Mismatch repair
MMRd	Mismatch repair deficiency
MMRp	Mismatch repair proficiency
MS	Microsatellite assay
MSI	Microsatellite instability
MSS	Microsatellite stability
NGS	Next-generation sequencing
NOS	Not otherwise specified
OS	Overall survival
PCR	Polymerase reaction chain
*wtKRAS*	Wild-type *KRAS*
