# Supplementary material for: KRAS Mutations in Colorectal Adenocarcinoma: Incidence and Association with Histological Features with Particular Reference to Gly12Asp in a Multicenter GIPAD Real-World Study
Source: Cancers (Basel). 2025 Aug 22;17(17):2721. doi: 10.3390/cancers17172721 (PMC12427293; doi:10.3390/cancers17172721)
Supplement: Supplementary file 1 [file cancers-17-02721-s001.zip › cancers-3700154-supplementary.pdf]

Table S1 Supplementary: Clinico-pathologic and molecular features of 2816 primary colorectal adenocarcinomas from 12 large Italian Surgical Pathology Units.

Centre	Centre 1	557 (19.8%)
	Centre 2	362 (12.9%)
	Centre 3	308 (10.9%)
	Centre 4	291 (10.3%)
	Centre 5	230 (8.2%)
	Centre 6	198 (7.0%)
	Centre 7	190 (6.7%)
	Centre 8	188 (6.7%)
	Centre 9	185 (6.6%)
	Centre 10	123 (4.4%)
	Centre 11	108 (3.8%)
	Centre 12	76 (2.7%)
Assay	NGS-bassed assay	1722 (61.2%)
	PCR-based assay	1060 (37.6%)
	MALDI-TOF	34 (1.2%)
Age[a]	>70 years	1472 (52.3%)
	≤70 years	1343 (47.7%)
Sex[b]	M	1627 (57.8%)
	F	1187 (42.2%)
Specimen	Biopsy	692 (24.6%)
	Surgical	2124 (75.4%)
Site[c]	Proximal	1011 (37.4%)
	Distal	483 (17.9%)
	Rectosigmoid	1208 (44.7%)
Histotype[d]	NOS	2323 (87.2%)
	Mucinous	247 (9.3%)
	Signet Ring cell	28 (1.1%)
	Medullary	20 (0.8%)
	Micropapillary	20 (0.8%)
	Adenosquamous	11 (0.5%)
	Undifferentiated	8 (0.3%)
	Serrated	5 (0.2%)
Grading[e]	High	853 (35.8%)
	Low	1531 (64.2%)
Budding[f]	Bd1	538 (38.0%)
	Bd2	398 (28.6%)
	Bd3	464 (33.4%)
Lymphovascular invasion[g]	No	642 (31.0%)
	Yes	1427 (69.0%)
Perineural invasion[h]	No	1066 (53.0%)

	Yes	947 (47.0%)
pT[i]	pT1	16 (1.0%)
	pT2	92 (5.9%)
	pT3	743 (57.4%)
	pT4	718 (45.8%)
	- pT4a	- 554
	- pT4b	- 155
	- na	- 9
pN[j]	pN0	446 (28.7%)
	pN1	609 (39.1%)
	- pN1a	- 221
	- pN1b	- 290
	- pN1c	- 91
	- na	- 7
	pN2	501 (32.2%)
	- pN2a	- 238
	- pN2b	- 250
	- na	- 13
MMR/MS status[k]	MMRd/MSI	436 (17.3%)
	MMRp/MSS	2082 (82.7%)

Abbreviations: MMR: Mismatch Repair; MMRd: Mismatch Repair deficiency; MMRp: Mismatch Repair proficiency; MS: Microsatellite assay; MSI: Microsatellite Instable; MSS: Microsatellite Stable.

Information not available for [a]1 case. [b]2 cases. [c]116 cases. [d]154 case. [k]298 cases
Information not available/assessable in [e]419. [f]1426 cases. [g]747 cases. [h]803 cases. [i]1249 cases. [j]1262 cases

Table S2. Supplementary: *KRAS* mutations frequency according to Age (A), Sex (B), Specimen (C), Budding (D), Perivascular (E) and Perineural (F) invasion, pT (G) and pN (H) stage, MMR/MS status (L).

		Gly12Asp				Gly13Asp				Gly12Val				Gly12Cys			
		Gly12Asp N=319 (25.5%)		*KRAS* non-Gly12Asp mut* N=934 (74.5%)		Gly13Asp N=243 (18.2%)		*KRAS* non-Gly13Asp mut N=1091 (81.8%)		Gly12Val N=234 (17.5%)		*KRAS* non-Gly12Val mut* N=1019 (82.5%)		Gly12Cys N=89 (6.7%)		*KRAS* non-Gly12Cys mut N=1245 (93.7%)	
**A** Age	>70 years	151	47.3%	475	50.9%	127	52.3%	542	49.7%	104	44.4%	522	51.2%	53	59.6%	616	49.5%
	≤70 years	168	52.7%	459	49.1%	116	47.7%	549	50.3%	130	55.6%	497	48.8%	36	40.4%	629	50.5%
	p value	0.278				0.466				0.061				0.066			
**B** Sex	M	187	58.6%	552	59.1%	135	55.6%	646	59.2%	146	62.4%	593	58.2%	53	59.6%	728	58.5%
	F	132	41.4%	382	40.9%	108	44.4%	445	40.8%	88	37.6%	426	41.8%	36	40.4%	517	41.5%
	p value	0.880				0.295				0.239				0.842			
**C** Specimen	Biopsy	89	27.9%	264	28.3%	67	27.6%	296	27.1%	65	27.8%	288	28.3%	30	33.7%	333	26.7%
	Surgical	230	72.1%	670	71.7%	176	72.4%	795	72.9%	169	72.2%	731	71.7%	59	66.3%	912	73.3%

	p value	0.900				0.889				0.882				0.154			
**D** Budding	Bd1	38	26.0%	151	35.3%	39	34.5%	164	33.1%	39	39.4%	150	31.6%	10	30.3%	193	33.5%
	Bd2	44	30.2%	116	27.1%	29	25.7%	139	28.0%	29	29.3%	131	27.6%	11	33.3%	157	27.3%
	Bd3	64	43.8%	161	37.6%	45	39.8%	193	38.9%	31	31.3%	194	40.8%	12	36.4%	226	39.2%
	p value	0.119				0.877				0.173				0.748			
**E**Lymphovascular invasion	No	69	31.1%	206	31.9%	53	31.4%	240	31.3%	55	34.2%	220	31.1%	19	33.3%	274	31.1%
	Yes	153	68.9%	440	68.1%	116	68.6%	528	68.8%	106	65.8%	487	68.9%	38	66.7%	606	68.9%
	p value	0.823				0.978				0.454				0.729			
**F** Perineural invasion[g]	No	119	54.3%	351	56.0%	91	57.6%	411	54.6%	89	56.3%	381	55.4%	28	48.3%	474	55.6%
	Yes	100	45.7%	276	44.0%	67	42.4%	342	45.4%	69	43.7%	307	44.6%	30	51.7%	379	44.4%
	p value	0.674				0.489				0.828				0.280			
**G** pT	pT1	3	1.7%	2	0.4%	0	0.0%	6	0.9%	0	0.0%	5	0.9%	0	0.0%	6	0.8%
	pT2	9	5.1%	25	4.9%	8	6.2%	30	4.7%	5	3.8%	29	5.2%	1	2.2%	37	5.2%
	pT3	83	47.2%	248	48.2%	56	43.4%	305	48.3%	70	53.4%	261	46.6%	23	50.0%	338	47.3%
	pT4	81	46.0%	240	46.6%	65	50.4%	291	46.0%	56	42.7%	265	47.3%	22	47.8%	334	46.7%
	p value	0.373				0.518				0.479				0.893			
**H** pN	pN0	46	26.3%	147	28.8%	36	28.1%	177	28.3%	39	29.8%	154	27.7%	12	26.1%	201	28.4%
	pN1	69	39.4%	224	43.8%	57	44.5%	264	42.2%	55	42.0%	238	42.9%	17	37.0%	304	42.9%
	pN2	60	34.3%	140	27.4%	35	27.3%	185	29.6%	37	28.2%	163	29.4%	17	37.0%	203	28.7%
	p value	0.223				0.853				0.896				0.482			
**L** MMR/MS status	MMRd/MSI	19	6.4%	72	8.6%	26	11.4%	67	6.8%	6	2.9%	85	9.1%	4	5.1%	89	7.9%
	MMRp/MSS	277	93.6%	766	91.4%	202	88.6%	914	93.2%	199	97.1%	844	90.9%	74	94.9%	1042	92.1%
	p value	0.237				**0.020**				**0.003**				0.380			

Abbreviations: MSI: Microsatellite Instable; MMRd: Mismatch Repair deficient profile; MMRp: Mismatch Repair proficient profile; MS: Microsatellite Status; MSS: Microsatellite Stable.

*n=82 G12X KRAS-mutated CRCs were excluded

Table S3 Supplementary: Missing data for variable and relative % of bioptic sample.

VARIABLE	TOTAL CASES WITH MISSING DATA (%)	BIOPTIC SAMPLES FOR MISSING DATA (%)
**AGE**	1/2816	0/1 (0%)
**SEX**	2/2816	1/2 (50%)
**ANATOMICAL SITE**	116/2816 (4%)	54/116 (46.6%)
**HISTOTYPE (according to WHO 2019)**	154/2816 (5.4%)	94/154 (61.0%)
**GRADING**	419/2816 (14.8%)	277/419 (66.1%)
**BUDDING**	1426/2816 (50.6%)	702/1426 (49.2%)
**VASCULAR INVASION**	747/2816 (26.5%)	675/747 (90.4%)
**pT**	1249/2816 (44.3%)	692/1249 (55.4%)
**pN**	1262/2816 (44.8%)	692/1262 (54.8%)
**MMR/MSI**	298/2816 (10.5%)	115/298 (38.5%)
